# Living a frozen life: a qualitative study on asylum seekers’ experiences and care practices at accommodation centers in Sweden

**DOI:** 10.1186/s13031-022-00480-y

**Published:** 2022-09-07

**Authors:** Charlotta van Eggermont Arwidson, Jessica Holmgren, Kristina Gottberg, Petter Tinghög, Henrik Eriksson

**Affiliations:** 1 Division of Nursing, Department of Neurobiology, Care Sciences and Society, Karolinska Institutet, Huddinge, Sweden; 2 Department of Health Sciences, Swedish Red Cross University, Huddinge, Sweden; 3 Department of Health Sciences, University West, Trollhättan, Sweden; 4 School of Health, Care and Social Welfare, Mälardalen University, Västerås, Sweden; 5 Division of Psychology, Department of Clinical Neuroscience, Karolinska Institutet, Solna, Sweden

**Keywords:** Accommodation centers, Asylum seekers, Care practices, Mental health, Peer support, Qualitative study, Wellbeing

## Abstract

**Background:**

Forced migrants fleeing conflict and violence face a high risk of mental health problems due to experiences before displacement, perilous journeys, and conditions in the new host societies. Asylum seekers seem to be in particularly vulnerable situations, indicated by higher prevalence rates of mental health problems compared to resettled refugees. Asylum seekers’ mental health is highly influenced by the conditions they face in host countries while awaiting a decision on their case. In Sweden, 40% of asylum seekers reside in state-provided accommodation centers during the asylum process. Collective accommodation centers for asylum seekers have been said to impose restrictive social conditions and to be associated with poorer mental health outcomes than other housing forms (e.g., self-organized housing). However, there seems to be a scarcity of qualitative studies exploring the experiences of asylum seekers in different contexts. The aim of this study was therefore to explore the experiences of asylum seekers and how they manage their mental wellbeing while living at accommodation centers in Sweden.

**Methods:**

Fourteen semi-structured interviews with asylum seekers were conducted at two accommodation centers in Sweden. Participants were recruited using purposeful sampling and represented a diverse group of asylum seekers regarding age, background, and gender. The data was analyzed using content analysis.

**Results:**

Three overarching categories were identified; 1) Frozen life, 2) Constant worrying and “overthinking”, and 3) Distractions and peer support**.** Participants experienced a state of being that could be characterized as a frozen life, which was associated with intense feelings of psychological distress, mostly described as manifesting itself in consuming patterns of ruminative thoughts, for instance overthinking and constant worrying. However, despite high levels of distress, participants demonstrated agency in managing negative mental health outcomes through self-care practices, peer support, and the development of care practices in caring for others in need.

**Conclusion:**

This study offers new insights into the everyday challenges that asylum seekers at accommodation centers face. Furthermore, it offers valuable observations of how asylum seekers at accommodation centers cope through self-care practices, peer support, and care practices in caring for peers in need. In order to enable sustainable and empowering support, mental health and psychosocial support services must identify and address both challenges and strengths, be grounded in the lived reality of asylum seekers, and build on existing resources. Moreover, further policy work needs to be done to enable faster asylum processes.

## Background

Globally, the numbers of people being forced to leave their homes due to conflict, violence, and persecution are higher than ever in human history [1]. There is an enduring concern and a substantial body of research highlighting the precarious mental health situation of those who have been forced to migrate [2–7]. Moreover, research has highlighted that asylum seekers seem to be in a particularly vulnerable situation owning to, among other things, an insecure legal status [6]. However, even though the experiences of asylum seekers most likely vary depending on the context, there seems to be a lack of studies that differentiate between asylum seekers based on this. Thus, the need to acknowledge and highlight the experiences and personal accounts of asylum seekers in different contexts continues to be of utmost importance.

While the United Nations (UN) Refugee Convention cites human beings’ right to protection, the interpretation and implementation of these rights depend on signatory states, and reception and protection conditions vary widely across countries [8]. Sweden has a long tradition as a destination country for many asylum seekers, and like many other countries, Sweden experienced an extraordinary inflow of refugees during the period 2015–2016, with a record high number of approximately 163,000 asylum seekers arriving in 2015 [9]. This can be compared with the years 2010–2011 when around 30,000 new asylum applications were received by the Swedish Migration Agency each year. The large number of people seeking protection placed great stress on the country’s receiving systems and triggered policy responses to reduce the inflow of people applying for asylum [10]. These legal changes were put in place as temporary provisions, but since the summer of 2021 the provision on, for example, the issuance of temporary protection permits to those granted asylum became a permanent part of the Swedish Aliens Act. The number of asylum seekers arriving in Sweden has significantly declined since 2015, with the country receiving only 11,410 asylum claims in 2021 [9]. Long waiting times continue to be a challenge in the Swedish asylum system, with the average processing time for a first-instance decision on an asylum claim being 271 days. However, with the possibility to appeal the decision, the process of receiving a final decision normally extends over longer periods of time, up to several years, and with great variation between people from different countries of origin. While awaiting the outcome of their asylum claim in Sweden, asylum seekers who cannot provide for themselves are offered housing by the Swedish Migration Agency, either at a large accommodation center or in apartments rented by the Agency, and are provided a basic allowance to cover their daily needs. Under certain conditions there are possibilities to apply for the right to work (e.g., if acceptable identity documents have been submitted and the asylum application is well founded) [9]. Additionally, adult asylum seekers are entitled to health care within the scope of “health care that cannot be postponed”, a somewhat contentious expression that has been criticized for being imprecise as to the content of such care, leaving the decision to individual care providers [11]. Children up to 18 years of age have the same entitlements as children permanently living in Sweden.

Being forcibly displaced is usually associated with extensive challenges, from pre-migratory experiences through perilous journeys to resettlement difficulties [7]. Additionally, many studies have highlighted that asylum seekers seem to be in a particularly vulnerable situation due to the uncertainty of the asylum process, indicated by higher prevalence rates of mental health problems compared to resettled refugees [6, 12–14]. Contemporary scholars have also pointed out that much of the earlier literature examining the mental health of asylum seekers and refugees has focused on pre-migratory trauma such as experiences of torture and other potentially traumatic events, but that in recent years the focus has shifted to exploring links between the resettlement phase and mental health [15, 16]. In line with this, a growing body of literature highlights a multitude of daily challenges in the asylum process that may adversely affect people’s health, both indirectly through mediating the effect of previous trauma and directly in its own right [13, 17–20]. These challenges include prolonged uncertainty regarding the outcome of one’s asylum claim, delays in processing the claim, constant fear of being returned to one’s country of origin, concern about family members back home, loneliness and boredom, prolonged feelings of insecurity and powerlessness, unemployment, and limited access to services [17, 19, 21]. The detention of asylum seekers has received specific attention in some studies, whose findings suggest that detention is particularly detrimental to mental health and inflicts long-term psychological harm to asylum seekers [22, 23]. Other studies have shown that asylum-seeking women are particularly at risk for poor mental health as they report higher psychological distress than asylum-seeking men [24]. Among other things, this has been linked to different gender roles that relate to different access to power and control over one's life between men and women [24]. Moreover, there is evidence indicating that asylum-seeking children are a particularly vulnerable subgroup [25]. Studies have also shown that the effects of being an asylum seeker seem to be cumulative, signifying that the duration of the asylum process has an overall impact on health; i.e., the longer the asylum procedure, the higher the risk for future mental health problems as well as hampered socioeconomic integration, given the importance of mental health for integration [17, 26, 27].

While the experiences of being an asylum seeker might substantially differ from one another, depending on contexts and living conditions, there seems to be only a small number of studies differentiating between groups of asylum seekers based on this. According to some researchers, this tendency risk creating a barrier to the development of robust situated knowledge on asylum seekers unique experiences [6, 28]. Though they are scarce, there are qualitative studies exploring the experiences of asylum seekers in different contexts, such as among community-based asylum seekers [29, 30] or at collective accommodation centers [31], which have been described as imposing more restrictive social conditions [29], being associated with poorer mental health outcomes [32], and as “unhomely” places triggering violent behavior among, for example, young adult male asylum seekers [33].

While there is a large body of literature focusing on prevalence rates of and risk factors for mental health problems among asylum seekers, as well as on their access to mental health care [34], less attention has been paid to protective factors or enablers for mental wellbeing in this group. Moreover, concerns have been voiced that focusing entirely on the aforementioned issues may contribute to a “medicalization of distress” and a common view of asylum seekers as a vulnerable and passive group, which risks ignoring the capacities that forced migrants bring with them [35, 36]. Though they are scarce, there are studies that explore the protective factors for asylum seekers, mainly focusing on the concepts of individual and/or community resilience as key factors for asylum seekers’ capacity to withstand experiences of trauma or distress, or as factors mediating the known risk factors effect on mental health of asylum seekers [28, 30]. In a recent systematic review from 2019, Posselt et al. [37] found that many studies focusing on protective factors for asylum seekers identified social support and religious/cultural-related factors as having a protective effect, followed by factors such as cognitive strategies, employment and economic activities, education and training, and behavioral strategies. The promotion of protective factors and enablers of health holds the potential to enhance mental health and prevent the development of mental disorders among asylum seekers, an important aspect to consider in the development of interventions designed to improve their mental health and wellbeing. Thus, this is an important aspect for scientific research to focus on. However, to the best of our knowledge, there seems to be a scarcity of qualitative studies exploring the subjective experiences of asylum seekers and how they manage their mental health concerns in different contexts. The aim of this study was therefore to explore the experiences of asylum seekers residing at accommodation centers in Sweden and their strategies for mental wellbeing, thereby contributing specific understanding and knowledge, much needed to promote and strengthen the mental wellbeing of asylum seekers.

## Aim

To explore the experiences of asylum seekers and how they manage their mental wellbeing while living at accommodation centers in Sweden.

## Methods and materials

### Design

This study used a descriptive qualitative approach as described by Sandelowski [38] to explore the experiences of asylum seekers. This approach is valuable for exploring areas where there is insufficient knowledge and a deepened understanding is warranted. Qualitative descriptions generate straight descriptions of a phenomenon in its everyday terms, especially of use for practitioners and policymakers [38].

### Setting and participants

The study was conducted at two of the largest accommodation centers in Sweden, housing 300 and 550 asylum seekers respectively (Swedish Migration Agency, personal communication, August 12, 2020). Both are in rural areas, a few kilometers outside larger cities, accommodating asylum seekers from over 50 countries. At the time of the study, approximately 40% of all asylum seekers residing in Sweden lived in housing facilities provided by the Swedish Migration Agency [9]. At the accommodation centers, asylum seekers were housed in rooms of two to six people by gender or family membership. Toilets, bathrooms, and kitchens were shared among several rooms, and the centers were based mainly on self-catering.

As shown in Table 1, the 14 participants represented a diverse group of asylum seekers, comprising six women and eight men aged 22–62 years. They were from eight countries and had different educational levels, from no formal education to a university degree. They had different family circumstances, with approximately half of the participants being married and three having children living with them at the center. Furthermore, their length of stay in Sweden at the time of the interviews varied from four months to nine years. The participants were also at various stages in their asylum processes: Some were awaiting the first-instance decision on their claim, while others had already received an initial negative decision and had appealed to the migration court.

**Table 1:** Sociodemographic information about the participants (n = 14)

	Description	Number of participants
Gender	Men	8
	Women	6
Age	20–30	5
	31–40	3
	41–50	3
	51–62 +	3
Marital status	Married living with partner	4
	Married not living with partner	2
	Single	7
	Widowed	1
Educational background	No formal education	2
	Primary	3
	Secondary	2
	University	6
Time in Sweden	< 6 months	1
	6 months–1 years	3
	1–< 2 years	1
	2–3 years	6
	> 4 years (max 9 years)	3
Country of origin	Sudan	1
	Iraq	3
	Syria	1
	Yemen	2
	Eritrea	4
	Ethiopia	1
	Pakistan	1
	Afghanistan	1

### Data collection

An interview guide was developed, which before data collection began was tested in pilot interviews with two asylum-seeking men recruited at a day center for migrants. After testing, minor changes were made to the interview guide. Pilot interviews were not included in the study results. Before recruiting participants, permission to conduct research was obtained from the managers of each accommodation center. In recruiting participants a combination of purposeful and snowball sampling techniques was applied, in an attempt to ensure a diversity of experiences with regard to gender, age, and educational background. This allowed us to explore the experiences of asylum seekers through a broad range of cases. The purposeful sampling was facilitated by local non-governmental organizations (NGOs) with access to the accommodation centers. Thus, asylum seekers engaged in activities or support programs run by local NGOs were approached individually, by volunteers from the same NGOs, with an invitation to participate in the study. All the volunteers involved in recruiting participants had been given written information about the study. In addition to the purposeful sampling, two study participants were identified with the help of other participants (i.e., snowball sampling). Data was collected via individual face-to-face, semi-structured interviews between September 2020 and March 2021 and were carried out on premises at the accommodation centers, provided by the local NGOs. The 14 interviews were conducted by the first author, a trained nurse with experience in clinical and research practice involving migrant health and cross-language issues, with Swedish as her native language. The interviews started with background questions about the participant’s family, education, and length of stay in Sweden. The questions that followed were designed to explore the two main areas following the aim of the study and included: Can you describe what it is like to be an asylum seeker? What do you do to promote your wellbeing? Additional probing questions were asked to deepen the responses and for clarity.

None of the participants involved in this study had English or Swedish as their native language. Interviews were conducted in two ways: in English between a bilingual Swedish-speaking researcher and a bilingual Eritrean/Ethiopian/Sudanese participant (n = 5), and between a Swedish-speaking researcher assisted by a professional interpreter via telephone (verbatim translation) and a participant in their native language (Arabic, Persian, Pashto) (n = 9). When an interpreter was used, instructions were given to the interpreter before each interview regarding the importance of translating everything, however small or seemingly nonsensical. The interpreters used were from well-known professional translation agencies.

The interviews were audio-recorded with permission from the participants, and lasted 35–79 min. Those conducted in English were transcribed verbatim by the first author. In the case of interviews assisted by interpreters, the recorded translations were transcribed verbatim by the first author. The number of interviews was guided by the concept of information power as described by Malterud et al. [39], considering the specificity of participants’ experiences (i.e., that the participants belonged to the target group while still showing some diversity in experiences), and the quality and information given in the interviews related to the aim.

### Data analysis

The data was analyzed using inductive qualitative content analysis as described by Patton [40]. The unit of analysis consisted of the 14 interviews transcribed into text.

As the distinction between data collection and data analysis is not absolute in qualitative research [40], the first stage of analyzing the data occurred during data collection and transcription, with the first author getting familiar with the data and noting various aspects of the content that were of importance to the aim of the study. The formal analysis began with the first and last authors, the latter a senior researcher in qualitative methods and nursing science, reading the transcripts independently several times to familiarize themselves with the data and gain a sense of the whole, writing comments, key words, and initial codes in the transcripts’ margins related to the overall research question; i.e., the experiences of asylum seekers at accommodation centers and their ways of managing their mental wellbeing. These two authors then discussed and compared similarities and differences in the initial coding to gain deeper insights. After this step, the interview transcripts were uploaded into the qualitative analysis software NVivo (release 1.4) to facilitate formal data coding in a systematic way. The first author then read the data several times, coding and recoding it during the process and naming the codes as close to the original text as possible. This step also included regular discussions with the last author to gain consensus on the coding scheme. In the next step, the process of identifying significant patterns and regularities in the coded data began, which in practical terms meant capturing and grouping similarities and differences in core experiences into categories. Throughout the process of developing categories, the data was read several times and the categories reorganized in order to ensure that they accurately represented the data and were as close to it as possible; i.e., with a low degree of interpretation. Final categorization was discussed among all authors and consensus was reached on its consistency, accuracy, and descriptive quality. The results are presented organized in categories, supported by quotations from the participants.

### Ethical considerations

Ethics and protection were considered in relation to the fact that asylum seekers, through their precarious legal status and limited access to services in Sweden, are in a particularly vulnerable situation. Before the interviews started, written information was provided to literate participants in Arabic and Persian, and all participants were informed verbally in a language they were proficient in, either in English or with the help of professional interpreters, about the purpose of the study and that their participation would not have any impact on their asylum claim. Written or verbal informed consent was obtained from all participants. Verbal consent was deemed necessary with participants who had low levels of literacy or were illiterate. Participants were informed that they had the right to withdraw their consent at any time during the interview and afterwards, without further explanation. It was important to ensure that the participants understood what consent and voluntariness meant, and a follow-up question was posed about this when necessary. In addition, to safeguard the participants’ mental wellbeing and in the event of participants being negatively affected or distressed by the interviews, a pre-planned distress protocol was used, designed to provide support during the interview and if needed to refer to mental health support services. On two occasions the distress protocol was used to refer participants to relevant support service. To preserve the participants’ confidentiality, the transcripts were anonymized and coded by number. In the presentation of the findings, all quotes are pseudonymized.

Ethical approval for this study was granted by the Swedish Ethical Review Authority (dnr. 2020–00,896).

## Results

From the data analysis, three qualitatively different categories related to the overall aim were identified. These were termed: 1) Frozen life, 2) Constant worrying and “overthinking”, and 3) Distractions and peer support.

### Frozen life

Participants experienced a state of being that could be characterized as a frozen life, that gave few possibilities to take control over one’s life situation. Nevertheless, when talking about different experiences related to life at the accommodation center, many of the participants were careful to express gratitude that life there provided security, warmth, and a roof over their heads. However, as some described, one of the core aspects of their experiences as asylum seekers was the overwhelming uncertainty about the future and the indefinite waiting time for a decision on their asylum claim. Many participants experienced this as a destructive and unhealthy process:

*“…but it’s difficult, because there’s no answer from immigration. There’s no timetable, you don’t know when you’ll have your interview, when you’ll have your answer. You’re staying here, you have no job, you can’t speak the language, you have nothing. Your future is like...you don’t see anything so that’s the thing. So I say most of the people here…I see people who have kids back home in their own countries who are sick. It hurts… […] And the whole situation isn’t healthy.” (Participant #1 -Sudan)*

Apart from describing the legal process of applying for asylum and the uncertainty characterizing it as causing distress, participants also described the environmental and social constraints as defining their experience living at the accommodation center. Many of them experienced that their lives at the accommodation center were geographically isolated and described that access to the wider host society was further limited by restricted public transport networks and prohibitive costs. Additionally, small financial resources as well as limited access to social networks in Swedish society or even skills to navigate a new society, including language, were described as adding to the experience of living a heavily confined life as an asylum seeker. In many of the narratives this was further illustrated through statements such as life being so heavily restricted that it could be compared with being a prisoner or as life having stopped. One young participant illustrated this, describing his life as being in a constant frozen state:

*“Being an asylum seeker, it’s like your life freezes for some moments. Not just moments but maybe years, you don’t know. Your life just freezes. You don’t know what’s going to happen to you. You can’t, me I read Swedish, but maybe after I get my decision, if it’s negative, I won’t use the Swedish anymore. So, it’s just like, your life freezes. Stops. And you just wait for your decision to continue your life.” (Participant #9 -Ethiopia)*

This experience of living a heavily confined and frozen life was further illustrated in narratives involving the feeling of being stuck and the fact that the only way forward was through the asylum process, making one’s whole life dependent upon the decision on the asylum claim, and leaving few possibilities to take control of or have any influence over one’s life situation.

Another aspect of the experience of a frozen life, shared by many of the participants, was that the resources and assets that used to constitute their sense of self had been lost or devalued; resources such as one’s education, profession, belongings, networks, family, and social status. In many interviews this was illustrated in statements such as being no one, being a changed person, or no longer being valued as a unique individual, further adding to the overall experience of a life that was stopped or frozen:

*“[I used to have] many options, because I have relationships with others, but nobody knows where I am. […] No one knows I’m here and that I’m nothing know. They think I have my company now…” (Participant #2 -Iraq)*

It was particularly common to describe the inability to work as something that deeply affected the overall experience of being an asylum seeker and one’s sense of identity and possibility to gain some measure of control over one’s life situation.

Some participants also expressed how a lack of privacy and sharing rooms and other facilities with people from different backgrounds added not only to the feeling of being limited but also to a feeling of insecurity about oneself, indicated by one of the participants talking about his experience of eating with unknown people in the cafeteria of the one accommodation centers:

*“I don’t know, sometimes if you put a sane person in a group of insane people, that sane person will probably become insane.” (Participant #8 -Eritrea)*

As part of the experience of being in a situation of a frozen life, sometimes for years, many participants also experienced having lost or even being deprived of important time in their life–time that under other circumstances would have been used to achieve life goals such as fulfilling a career plan, studying, working, being with one’s children, contributing to society, or starting a family.

*“I am, for example, 38 years old. I don’t know what will happen to me. I have no family of my own, I have no children, I have nothing to think about. Everything is stopped. During this time, you wait, you can do nothing, you’re hopeless. You can’t fall in love, you can’t be in a relationship with someone, you can’t do anything at all, everything is stopped.” (Participant #14- Iraq)*

Some expressed that this experience of loss and of being deprived of time to live a life according to one’s personal life goals entailed irreversible losses that they experienced had the potential to impact their future life in a negative way.

### Constant worrying and “overthinking”

The experience of life as an asylum seeker, living a frozen life, was spoken of in almost all interviews as a source of constant worrying and overthinking, with overthinking described as negative thought patterns, a state of relentless rumination, that consumed the mind. This shared experience of worrying and overthinking also shared similarities in content, such as worrying about the future and what would happen if one was deported, or dwelling on how one had worded things or the right things to say during interviews with the Migration Agency. Apart from the common patterns of overthinking the possible outcomes of one’s asylum claim, there were also individual differences in the content of thoughts. One of the elderly participants, an illiterate woman, expressed that in addition to worrying and constantly thinking about her asylum claim, she was also experiencing constant worries and fears about her own safety moving around and navigating the new surroundings at the accommodation center. Another factor that seemed to fuel the constant rumination was separation from and uncertainty about one’s family. One participant spoke of her relentless daily questioning of herself for leaving her four children behind and her constant worry about their wellbeing. Being a parent and living with children as an asylum seeker also seemed to add worries over the children’s wellbeing and the possibility to provide for them with limited resources. One participant, who had been subjected to difficult traumas in their home country, indicated that the situation of uncertainty and waiting indefinitely, along with the feeling of insecurity at having to live with unknown people at the accommodation center, aggravated the constant re-experiencing and recalling of these traumas.

Many participants experienced that this state of constant worrying and overthinking had seriously harmed both their physical and mental health. One participant illustrated how the situation as an asylum seeker and the relentless rumination inevitably affected her mental health:

*“You already don’t know, your situation isn’t stable, you aren’t stable […] you start to feel nervous. You start to overthink the things and then you’re going to get into a depression whether you want to or not.” (Participant #5 -Eritrea)*

Other participants spoke of how constant rumination had affected their physical health and believed it had caused diabetes or impaired vison. To be in this state of overthinking was also reported to cause sleepless nights and extreme tiredness. One participant, who had undergone several years of indefinite waiting for a decision on his and his family’s asylum claim, reported that his brain was so tired of all the thoughts going around in his head that he experienced feeling indifferent to what was happening around him, as well as feeling emotionally cold inside:

*“I don’t feel like I have that energy anymore. My brain is tired after all the thinking and pondering. […] I feel a bit like I’ve become cold and indifferent, and just feel like I have no feelings anymore. I have no feelings left, I just let the days pass.” (Participant #4 -Afghanistan)*

In line with this experience, some participants also expressed worries that this period of feeling distress would have long-lasting consequences on their health and wellbeing, raising concerns about what would become of them:

*“So when you have this long waiting time, you lose hope, you become hopeless. So you lose hope that you can have a normal life in the future.” (Participant #14 -Iraq)*

Regardless of the outcome of the asylum process, this participant feared that the experience of being an asylum seeker would have a harmful impact on their possibility to live a “normal” life even after getting a decision.

Several participants also voiced the experience that adding to the burden of constant rumination was the fear of sharing these thoughts with others, not wanting to be seen as week or abnormal. Some participants described that they pretended to feel fine although they were not:

*“I always try to show that everything’s good and that everything’s okay with me even in front of the people who are close to me and who see me every day, but really it’s a nightmare I go through.” (Participant #2 -Iraq)*

Moreover, some of the participants associated this experience of being afraid to share one’s worries and thoughts with an increased feeling of loneliness.

### Distractions and peer support

Participants described different ways of managing their mental wellbeing in everyday life. In general, everyday life at the accommodation centers was described as boring and focused on keeping busy to distract oneself from worrying thoughts and overthinking. Some of the narratives indicated a very monotonic and passive life, mostly spent in bed, distracting oneself with a cell phone or laptop, scrolling through social media, taking contact with family and friends in other parts of the world, or searching for available online courses, interrupted only by meal breaks and chats with roommates. One participant described the free Internet as a blessing for passing the time and keeping one’s mind focused on something besides the negative thought patterns in one’s head. It was not uncommon for these participants to also describe changed sleep patterns whereby they usually slept during the day and lay awake at night:

*“After that, when I sit with my phone and check the phone and I do nothing else until three or four in the morning, then I can sleep a little. And the next day it’ll be the same and the same and the same.” (Participant #14 -Iraq)*

Other participants described everyday life as centered around daily chores such as cooking, buying groceries, washing clothes, or preparing children for school, which provided a certain routine in daily life, which seemed to offer a distraction from mental distress.

In discussing specific tactics for dealing with mental distress and promoting wellbeing, there were stories about self-medication with alcohol and drugs as well as using prescription drugs to cope with symptoms of mental distress. Others related that religion and prayer were important for getting by. In many of the narratives, self-care activities such as taking a long walk, going to the gym, or reading books were reported to be distractions for the mind:

*“I try first and foremost to distract myself, either to read a book or go out or do something that distracts me. I try to do everything to avoid thinking about the problems I have.” (Participant #6 -Yemen)*

However, the most common factor said to offer relief from mental distress was social interaction and support from other asylum seekers. Talking to others who were in the same situation created feelings of emotional support and of not being alone with this experience, as illustrated below by one participant:

*“It helps to feel that you’re in a family, among people who care about you; that they’re sharing your things.” (Participant #5 -Eritrea)*

This, as well as sharing information and advice, were described as important parts of the peer support received from other asylum seekers. Many of the narratives also illustrated how this mutuality in the situation created a concern for others’ wellbeing and a sense of caring for each other. Participants described that they tried to motivate others to leave their room, go out and move around, or talk about how they felt, or that they tried to distract others by engaging in conversation about ordinary things like football:

*“But this is what I see, but I see people sometimes having a bad day, yeah so, we try to engage them in a conversation, that’s it, yeah we try to...yeah he’s not feeling good today, then the next day he can come up with some energy” (Participant #11 -Eritrea)*

One young participant told of a group of asylum seekers at the center who often organized social activities to ease their minds:

*“…like yesterday when we gathered in the room, we felt so ehh we were down, but we didn’t show that we were down. When I just told them that, let’s make popcorn, all of them they got excited. Okay, I’ll make this and I’ll make this and then they put music on. They were dancing like crazy. Truly, they got all the negativity out.” (Participant #5- Eritrea)*

Another aspect of this concern and caring for each other was a sense of caring for others in need. Several participants talked about worrying behaviors in others, such as isolating themselves in their room, not talking to anyone, or being sad all the time, often in conjunction with a negative decision on their asylum claim. Especially one participant told of how a sort of crisis management system had been developed among some groups of friends within the accommodation center.

“Y*ou surround that person, and you try to give them comfort... […] So you don’t leave that person alone. At least for a week or something. You eat with them, you cook with them, sometimes you sleep together with them. You go to xx [city name] with them or other places. And then after a week, they adapt.” (Participant #8 -Eritrea)*

Through this support system comfort, and advice was provided to the person in need until they had adapted to the new situation.

When talking about the importance of social support and interactions, many participants also described how local NGOs facilitated gatherings and provided possibilities for social participation, not only through organizing activities but also through providing possibilities to engage as volunteers. Serval participants spoke of how this kind of engagement created a sense of connectedness and satisfaction at doing something meaningful. However, some participants also mentioned experiencing barriers to social interaction. One elderly woman, only proficient in her native language, described that language barriers prevented her from interacting with other asylum seekers and consequently enhanced her feeling of being alone with her worries. Furthermore, a participant who had been subjected to difficult traumas in the home country also described how mental illness had become a barrier to engaging in social networks and interacting with others at the accommodation center.

Moreover, in the different narratives involving managing mental distress in everyday life, it seemed as if duration of stay had an overall impact, pointing to different phases in ways of dealing with distress. One participant, who also had one of the longest durations of stay at the accommodation center, talked about how he had initially been active and engaged but that he felt he had changed with time:

*“I’ve been active, it’s true, but over time you get dead inside yourself as well. This long time of not knowing what will happen in the future, you don’t know the important things, you know nothing, and you can’t plan your future […] I would say this, time can be a killer; that is, when you don’t get your decision, when time goes by year after year and nothing happens. You stay in your place and wait, it’s very deadly.” (Participant #14 -Iraq)*

Extended time spent waiting eventually demotivated participants to engage and be active, rendering activities meaningless, and for some created the feeling that nothing makes a difference when it comes to managing mental distress and changing the situation.

## Discussion

The aim of this qualitative study was to explore asylum seekers’ experiences of living at accommodation centers in Sweden and their ways of managing mental wellbeing. The findings show that the participants experienced a state of being whereby they described life as hardly any life at all, or as being “stuck”, frozen in time and space. This state of a frozen life was shaped by the experiences of living with uncertainty about the future, together with the constraints and losses that life at the accommodation center imposed on the participants. Experiencing this state of being, the participants described feeling that they were powerless and lacked control over their life. Furthermore, it contained an aspect of feeling deprived of time to live a meaningful life, an aspect tied to the temporal dimension and the duration of the asylum procedure. Altogether, the experience of a frozen life was associated with an everyday life characterized by intense feelings of psychological distress, mostly described as manifesting itself in consuming patterns of ruminative thoughts, for instance overthinking and constantly worrying. However, despite high levels of distress, participants described how social support between asylum seekers brought relief from rumination, and how they demonstrated agency in managing negative mental health outcomes through self-care practices, peer support, and the development of care practices, specifically showing compassion and caring for others in need.

Asylum seekers all over the world go through periods of waiting for a decision on their asylum claim. Much is at stake in this decision: Either protection or expulsion awaits in the future, and the experience of embodying both these possible outcomes simultaneously for undetermined lengths of time forms an essential part of the everyday life of asylum seekers. In previous literature, different concepts are used to describe this experience, the most common being “living in limbo” or “existential limbo” [41, 42]. The category of a frozen life is comparable to this concept. However, the concept of limbo tends to refer to the indeterminate legal state held by asylum seekers, while the concept of a frozen life might have a broader approach, referring to a state of life in which life has been frozen in a certain place and time, and as such also defined by environmental and social factors (e.g., living geographically isolated, financial difficulties, lack of social networks, language barriers). As a concept, frozen life also shares similarities with how Margherita and Tessitore refer to the characteristic of the asylum seekers’ status as an identity fracture [43].

When it comes to the important feature of living with uncertainty and its detrimental effects on asylum seekers’ mental health, this has been highlighted in previous literature [17, 32, 41]. Moreover, it has been suggested that the experience of living with uncertainty might impact help-seeking behavior and engagement with therapeutic interventions in a negative way for asylum seekers [41, 44]. Following a more conceptual discussion, the concept of *living with uncertainty* has received some attention from different disciplines, highlighting the importance of understanding the influence of uncertainty on people’s everyday lives, on their actions and reactions [45]. In a concept analysis from nursing scholar Janice Penrod [45], it is suggested that there are different modes of uncertainty, whereby the nature of the experience of uncertainty is determined by a person’s sense of control and confidence in the situation. Drawing from this conceptual framework and by considering the experiences expressed by our participants about having hardly any control over one’s life circumstances or lacking confidence in the situation, our findings might point to an experience of living with uncertainty in its most discomforting, overwhelming, and immobilizing mode. Thus, it underscores the critical importance of considering the impact of living with uncertainty on asylum seekers. Furthermore, as the category of a frozen life implies, the uncertainty must be considered in relation to the multitude of challenges that face asylum seekers in their everyday life. Throughout the narratives in our study, it is highlighted how constraints and losses in the current situation interact in shaping how the asylum seekers experience their everyday life, and by extension their mental health. This finding is in accordance with research highlighting how the post-migration context influences the mental health of asylum seekers [16, 19, 21].

Another key finding in this study was the descriptions of how the participants experienced the psychological distress caused by the state of living a frozen life. According to them the most common manifestations of distress are ruminative thought patterns, “overthinking”, and worries. These manifestations are present in almost all the participants’ narratives as a pervasive and overwhelming experience, perceived as gradually consuming their wellbeing. This kind of manifestation of psychological distress, i.e. overthinking, has been described in an earlier qualitative study exploring asylum seekers’ mental health experiences, providing support for the relevance of our findings [46]. Additionally, as an idiom of distress, overthinking (or the closely related expression “thinking too much”) is a phenomenon that has been described in previous literature as a common way of communicating distress in local and culturally specific ways [47–49]. Kaiser et al. [48] describe “thinking too much” as an expression that can be associated with clinical constructs of psychiatric illness, as well as an idiom for conveying suffering in a more general, non-pathological way, related to one’s social position and perceived vulnerability. Our study population was non-clinical, and our aim was to explore subjective experiences; whether or not the experienced distress should be interpreted as a symptom of psychiatric diagnosis is not within the study’s scope. However, the findings suggest that the participants themselves experienced the psychological distress as a pathway to ill health, both physical and mental, indicating that they experience that the psychological distress has cumulative effects on health; i.e., the longer the time spent waiting, the larger the impact on one’s health. These findings also echo previous studies that have shown that longer waiting times for protection are associated with poorer mental health outcomes, consequently also increasing the risk of harming the individual’s ability to integrate in the host country once protection is granted [17, 20]. In addition, our findings indicate that asylum seekers have concerns about long-lasting effects of the current situation on their future life, further adding to a vicious circle of emotional distress.

An important aspect in identifying idioms of distress concerns addressing stigma surrounding mental health. Kaiser et al. [48] describe that mental health communication using medicalized concepts risks unintentionally contributing to stigmatization, and that drawing on local idioms might help reduce stigma and promote mental wellbeing.

One of the most interesting findings in the current study concerns how the participants describe tactics and ways of managing the heavy burden of psychological distress associated with everyday life and ways of promoting mental wellbeing. The overall findings suggest that everyday life is characterized by inactivity and limited agency, seeking ways to distract from worrying thoughts, even though this was experienced as challenging due to a lack of resources. Furthermore, participants describe how social support plays an integral role in their coping with distress, in both an emotional and an instrumental way. This is in accordance with existing literature addressing coping and resilience among asylum seekers, in which social support has been identified as vital for maintaining mental wellbeing [37]. What is less studied is the care practices that participants say develop in this situation of shared experience. For example, previous literature has illustrated how life at collective accommodation centers can trigger violent behavior among certain groups [33]. However, that the mutual experience of living together at accommodations centers also has the potential to trigger networks of peer support and care practices has not been described in detail. In contrast, in a study from the Netherlands it was observed that, due to the burden of multiple challenges in everyday life, asylum seekers’ capacity for mutual concern and support were limited [50]. In our study, the participants’ narratives illustrate how living together at the accommodation center meant gaining invaluable understanding and knowledge for identifying worrying signs and behaviors in peers and learning how to pay attention to others’ wellbeing. This in turn developed into caregiving practices, such as offering distractions, trying to motivate exercise, or providing companionship. These findings illustrate that, in addition to negative mental health consequences in reaction to the harmful asylum experience, there are other possible responses to this situation. Some participants were able to also transform the adversities into caring for others. Furthermore, these experiences existed simultaneously rather than being mutually exclusive, which underscores the complexity of asylum seekers’ experiences and reminds us not to simplify the experience into one-sided perspectives of either suffering or resilience. The narratives suggest that these caring practices exist mostly within groups who share a common cultural and linguistic bond, and within groups of the same gender. There might also be a temporal dimension related to the findings, suggesting that the duration of waiting wears out the motivation to engage in activities. However, this requires further investigation to be explored in more depth. As a related aspect of this caring for others, some participants described that volunteering with local NGOs gave them the opportunity to do something for another person, which created a feeling of meaningfulness. Volunteering and activism as coping strategies have been studied in wider refugee groups, i.e. asylum seekers and resettled refugees, and have been shown to be strategies that help people to find meaning in suffering and to avoid occupying the role of simply a victim [36, 51]. Our findings seem to underscore this research. Undoubtedly, this finding is important as it highlights the agency asylum seekers can demonstrate despite their personal suffering within the constraints of the accommodation centers. This finding aligns with what Papadopoulos framed in an article from 2007 [52], in which he emphasizes the general tendency of refugees to survive adversity, who with reasonably facilitative conditions and resources often can and do manage with minimal assistance. He calls this adversity-activated development. There are also studies advocating peer support as a crucial lifeline for asylum seekers and as essential in improving their wellbeing [53]. Furthermore, this finding highlights the importance of identifying not only the challenges asylum seekers face but also their collective and individual strengths and abilities, along with the caregiving structures within the actual community to promote wellbeing in a sustainable and empowering way.

In summary, being an asylum seeker puts a heavy toll on one’s mental health and is experienced as a pathway to ill health. The overall implications of this finding point to an urgent need for support to promote the mental wellbeing of asylum seekers. Additionally, the findings suggest that asylum seekers cite as the cause of their distress a complex and dynamic interplay between individual, social, and environmental factors, calling for a systematic approach in assessing the complexities behind the mental health outcomes. In line with this, there are various frameworks or models proposed for use in assessing sources of distress that provide a holistic account of the refugee’s experiences and consequently direct interventions to target factors at multiple levels. For instance, researchers have advocated a social ecological model that addresses ongoing resettlement stressors at the individual, family, community, and society levels as well as factors in pre- and post-migration contexts [54]. In light of our findings, it might be argued that such models would help to broaden the understanding of what affects asylum seekers’ mental health, which furthermore would be necessary to inform interventions directed at promoting mental health. There are other multi-layered approaches targeting factors at all these levels to respond accurately to the mental health concerns of asylum seekers. The guidelines developed by the Inter-Agency Standing Committee (IASC) on Mental Health and Psychosocial Support in Emergency Settings [55] also provide a holistic account of promoting a phased approach to interventions. The guidelines promote a multi-layered system of support, integrating non-specialized activities designed to strengthen protective factors (for example, strengthening informal social support and self-care practices) and prevention measures, with specialized support for those in need of it. This implies that although there are individuals in need of individualized treatment, the situation calls for more than simply improved health care, emphasizing the importance of a supportive environment and a reduction of daily stressors, which is supported by our findings. To achieve such integrated and multi-layered support there is also a need for coordinated actions and partnerships between various actors. In our study, the role of civil-society actors was identified as important in promoting psychological wellbeing through offering opportunities for meaningful activities and volunteering. NGOs served as a vital complement to other support systems, and were sometimes described as the asylum seekers’ only contact with the wider host society and as bridging the gap between them and society. This unique role of NGOs also points to a need in future research to further explore the implications of the part they play in promoting mental health.

Moreover, our findings highlight the importance of identifying and acknowledging the strengths and resources within the actual community of asylum seekers. In engaging with asylum seekers, it is vital to contextualize and give attention to how they describe their experiences of distress and suffering and what they believe alleviates the distress. Consequently, the findings might also highlight the relevance of addressing and challenging stigmatizing labeling and assumptions regarding asylum seekers, such as seeing them as suffering passive bystanders, in areas like policy, research, and service provision, which risks ignoring the unique capacities of affected populations [56]. Beyond the distress and suffering, this study identified narratives that described how the asylum seekers were gaining new knowledge related to mental health and the development of caring practices, which may be linked to the mutuality of the experience. Interventions designed to promote mental health should have an inclusive approach and support asylum seekers’ own initiatives to promote mental wellbeing, such as self-care and peer support activities and caring practices.

Finally, the temporal dimension forms an essential part of asylum seekers’ experience, i.e. long waiting times, implying that targeting factors at policy level such as advocating for faster and more transparent asylum processes might be vital in efforts to promote asylum seekers’ mental health. In accordance with this analysis, previous studies illustrate that asylum seekers perceived permanent legal status to be the most effective form of intervention for mental health concerns [41]. Another study found that the legal change from being an asylum seeker to receiving permanent legal status was associated with significant improvements in mental health [57]. Subsequently, researchers from all over the world have called for faster and more transparent asylum processes [6, 17, 26, 58], and this study adds to the weight of these calls. However, researchers have also portrayed the situation as entailing state-sanctioned policies of uncertainty [20, 59], highlighting that there might be conflicting interests between regulated migration and health-supporting policies for asylum seekers. This might suggest a dilemma in reconciling the tensions between policies of regulated migration with providing supportive resources for asylum seekers, an issue that warrants further investigation in future research.

## Strengths and limitations

The findings here need to be considered in light of some limitations, the most important being that the study only presented the views of a relatively small and heterogeneous sample of asylum seekers in Sweden and thus might not capture the full experience of being an asylum seeker at an accommodation center in Sweden. Subsequently, the findings may not be transferable to other populations of asylum seekers. In addition, the small sample did not offer the possibility to investigate differences in findings by, for example, gender, country of origin, age, or length of stay in Sweden, which are undoubtedly factors that in different ways shape the experience of being an asylum seeker. Furthermore, to be able to identify more culturally specific experiences, we would have needed to focus more on a particular subgroup of asylum seekers. There are also limitations related to the methodological challenges in conducting cross-language research [60]. Some of the interviews were conducted with the help of interpreters and details and nuances may have been lost in the translation. In addition, the presence of a third person may have influenced the responses of the participants. However, this was perhaps mitigated by the fact that the interpreters participated via telephone. There is also a potential limitation when it comes to interviews with bilingual participants in English: Unless both parties are completely proficient in English, this can result in poor accounts of their experiences, which may have affected the results in this study. However, the benefits of being able to give voice to people who are likely limited by language barriers and therefore potentially have unmet needs likely outweigh the limitations [60]. Furthermore, although this could possibly be seen as a strength, a single-researcher approach with the same researcher conducting the interviews, transcriptions, and analysis can open up for personal bias in the study, grounded in the researcher’s prejudice and attitudes; therefore, it is regarded as a limitation [40]. However, through including the co-researchers in all steps in the analysis process, the first author’s personal bias was reduced; the team of researchers has also critically reflected over these issues throughout the research process. Despite the limited generalizability of the small and heterogenous sample, the main benefit of the findings is the new insights into the experiences and capacities of asylum seekers at accommodation centers to be built upon in future studies.

## Conclusion

This study has described how asylum seekers at accommodation centers in Sweden experience the asylum process and everyday life at the centers as a pathway to poor mental health, with a risk of long-lasting mental health consequences. The findings confirm previous research that has identified asylum seekers to be in particularly vulnerable situations due to the precarious reception conditions at the accommodation centers provided by receiving countries. However, simultaneous with the experience of a precarious state of being as an asylum seeker, the study also showed that the experience triggered caregiving networks within the community of asylum seekers at the accommodation centers, highlighting the importance of not simplifying the experience into one-sided perspectives of either suffering or resilience. The findings suggest a more holistic and context-centered approach to the mental health and wellbeing of asylum seekers, not only when it comes to assessing the needs and abilities within the community but also in promoting interventions directed at asylum seekers. In addition to individualized treatment options, promotional and preventive measures that focus on a supportive environment and the reduction of daily stressors are greatly needed. Moreover, interventions should have an inclusive approach and support asylum seekers’ own initiatives to promote mental wellbeing and care for each other. Our findings draw attention to the importance of peer support and care practices developed by asylum seekers to care for people in need, as well as the importance of meaningful and empowering activities. Finally, to reduce the risk of long-lasting mental health problems, we cannot overlook the effect of the temporal dimension and the health impact of protracted waiting for a decision on one’s asylum claim. Asylum procedures should be fast-tracked to minimize the cumulative effects of the psychological distress which otherwise might further undermine asylum seekers’ life chances in both the short and the long term.

## Data Availability

In order to protect the participants’ privacy, the dataset generated during the current study is not publicly available. It is, however, available from the corresponding author on reasonable request.
